# Exploring New Horizons for Wine Grapes: Modulating Functional Effects by Varying Harvest Timing and Solar Exposure

**DOI:** 10.3390/foods13060857

**Published:** 2024-03-12

**Authors:** Daniel Rico, Daniel Schorn-García, Laura Aceña, María Jesús García-Casas, Olga Busto, Ricard Boqué, Montserrat Mestres, Ana Belén Martín-Diana

**Affiliations:** 1 Department of Medicine, Dermatology and Toxicology, Universidad de Valladolid, Av. Ramón y Cajal, 7, 47005 Valladolid, Spain; daniel.rico@uva.es; 2 Chemometrics and Sensorics for Analytical Solutions (CHEMOSENS) Group, Department of Analytical Chemistry and Organic Chemistry, Universitat Rovira i Virgili, Campus Sescelades, Edifici N4, C/Marcel⋅lí Domingo 1, 43007 Tarragona, Spain; daniel.schorn@urv.cat (D.S.-G.); laura.acena@urv.cat (L.A.); olga.busto@urv.cat (O.B.); ricard.boque@urv.cat (R.B.); montserrat.mestres@urv.cat (M.M.); 3 Agrarian Technological Institute of Castilla and Leon (ITACyL), Ctra. Burgos Km 119, Finca Zamadueñas, 47071 Valladolid, Spain; garcasmj@itacyl.es

**Keywords:** sun, shade, ripening, pomace, juice, grenache, cabernet sauvignon, total antioxidant capacity

## Abstract

Grenache (GN) and Cabernet Sauvignon (CS) are two traditional red grape varieties widely cultivated in the Mediterranean area and both late-ripening cultivars, which makes them less sensitive to global warming conditions and more stable to harvest timing. Although different studies have evaluated the final antioxidant properties of grapes and pomaces, few studies have explored the effect of sun exposure and harvest on the nutritional and antioxidant properties of these products. This study investigates the control of sunlight and ripening as tools to tailor nutritional and antioxidant properties of grape juices (GJ) and their byproducts (pomace GP). The compositional analysis showed no significant (*p* ≥ 0.05) differences associated to either harvesting timing or exposure to sunlight for either of the two studied varieties. However, differences (*p* ≤ 0.05) were observed between varieties of protein and total dietary fibre (TDF). CS protein content ranged from 0.52 to 3.88 (g 100 g^−1^) in GJ and from 1.0 to 1.32 (g 100 g^−1^) in GP; meanwhile, GN had higher protein values in GJ (from 2.11 to 4.77 g 100 g^−1^) and GP (from 5.11 to 6.75 g 100 g^−1^). The opposite behaviour was observed in TDF; CS grape had higher values for juice (from 11.43 to 19.53 g 100 g^−1^) and pomace (from 42.20 to 65.80 g 100 g^−1^) than GN (from 11.43 to 17.22 g 100 g^−1^ in juice and from 25.90 to 54.0 g 100 g^−1^ in pomace). The total phenolic content (TP) in GP was 100 times higher than in the juices and showed a much less pronounced evolution compared to the GJ during the harvesting time. GN TP values ranged from 5835 to 8772 mg GAE 100 g^−1^; meanwhile, CS values ranged from 7637 to 9040 mg GAE 100 g^−1^. A significant (*p* ≤ 0.05) correlation between the TP total antioxidant capacity (TAC) results was observed, regardless of variety, harvesting time, and sunlight exposure. These findings show how the control of different factors can contribute to obtain modified grape-derived products from conventional varieties beyond the wine market.

## 1. Introduction

Grapes are the largest fruit crop worldwide and hold a multifaceted and influential role globally, impacting economies, cultures, tourism, and trade. Its significance extends beyond economic considerations, encompassing social and environmental dimensions, making it an important component of the global primary production and processing industries. This is why the wine sector, recognising the broader implications of its practices, has emerged as one of the pioneers in adopting greener strategies over the last decade [1,2]. Despite these advances, the wine industry continues to generate substantial volumes of waste, particularly grape byproducts, with over fourteen million tonnes produced annually in Europe alone [3]. This waste, which consists of 75% wastewater [4] and 25% biodegradable solids like pomace, stems, and leaves [5,6], contains valuable nutrients such as polysaccharides and bioactive compounds, notably polyphenols. The composition of these byproducts varies considerably depending on the grape variety and agronomic practices [7,8].

Grape composition reflects the outcome of many physiological and biochemical interactions between the grape variety and its environmental conditions (soil, topography, and climate, among others) [9]. Temperature, sunlight exposure, and precipitation can affect grapes’ growth and ripening, berry composition, and primary and secondary metabolites such as phenolics and volatiles affecting the antioxidant properties and the quality of the final products [10,11].

The antioxidant potential of grapes is closely linked to their phenolic content and their chemical structures, influencing the capacity to reduce reactive oxygen species (ROS) and chelate metal ions. Polyphenols are compounds with a high ability to donate protons and/or electrons, and their radical intermediates are relatively stable due to the resonance delocalisation of the unpaired electron within the aromatic ring, and the lack of suitable positions for the attack caused by molecular oxygen [12]. The polyphenolic composition in grapes and grape byproducts is complex, as it comprises flavonoids such as flavonols, flavanols, and anthocyanins, and non-flavonoids, including phenolic acids and stilbenes. Furthermore, this complexity is heightened by the fact that their concentration varies and is distributed differently in the different specific fractions of the grape fruit, as is the case with anthocyanins, which are mainly found in the skins of red grapes. These compounds not only provide colour to the fruits, but also protect them against light radiation (UV) and display biocidal effects against bacteria and fungi [13].

It can be stated that winemaking byproducts, including grape pomace, offer a promising natural source of antioxidant additives, showing significant possibilities in contrast to the use of synthetic antioxidants, which are currently under scrutiny due to concerns about their potential toxicity [14]. In fact, for instance, tartaric acid and enocyanine (derived from anthocyanins in grape skin) have already been permitted by the EFSA (European Food Safety Authority) as food dye in beverages, marmalades, candies, ice creams, and pharmaceutical products.

Additionally, many studies have revealed that the addition of grape pomace to a wide range of food products, such as plant, dairy, and meat products, improves their nutritional value [15]. For example, grape pomace has been used to increase the content of dietary fibre and polyphenols, as well as to improve antioxidant activity in baked goods, such as muffins, biscuits, bread, cookies, and extruded cereals, among others [16]. Moreover, some studies have shown that fortification with grape byproducts could inhibit lipid oxidation and extend the shelf life of processed foods [17,18].

Among the different grape varieties, ‘Grenache’ and ‘Cabernet Sauvignon’ are two traditional ones widely used in the wine industry. These are late-ripening cultivars, making them less sensitive to global warming conditions and ensuring more stable harvesting timing [19]. Different studies have focused on the physiological aspects responsible for the quality and sensory characteristics of grapes. However, there is scarce information available on the evolution of their composition or their antioxidant activity during ripening. Acquiring this information would prove very useful from an oenological standpoint, as it would facilitate the production of wines with an optimal concentration of polyphenols, thereby ensuring their stability. Furthermore, given the increasing emphasis on the reuse and valorisation of byproducts to attain sustainable production practices, possessing this information would also enable us to obtain juices and pomaces with a specific polyphenol concentration, thus endowing them with heightened functional properties.

During the last few years, the European Commission has defined different strategies to promote sustainable development, among which a circular economy action plan (CEAP) was adopted in 2020 [20], with the aim to contribute to the main goals of the European Green Deal [21]. The adoption of circular economy principles has a broader scope than isolated actions to reduce energy, water and gas consumption, and promote waste recycling. The CEAP promotes initiatives along the entire production life cycle, targeting how products are designed, favouring circular economy (CE) processes, and promoting sustainable consumption, aiming for a holistic bio-economy system [22].

In this sense, the CE has gained great importance globally, but especially in the food processing industry. Unlike linear production models, the circular economy prioritises sustainability, minimising waste and making the use of new resources more available. In the processing industry, adopting CE principles for the transformation of raw materials into finished products may result in various benefits. These are associated with an efficient use of resources, emphasising recycling and remanufacturing, promoting the design of novel products through new technologies that support circularity, and leading to more sustainable and competitive industrial operations [23]. Adherence to CE principles in the processing industries can help the compliance with future and more restrictive regulations and avoid legal issues.

Finally, it must be considered that there is a need for the implementation of new strategies in the circular economy (CE) to reduce the impact of wine industry processing, including adaptive measures to deal with unpredictable weather, droughts, and rising temperature. In order to increase the efficiency of the use of the grape fruit, while favouring CE strategies through byproduct valorisation, the objective of this work was to gain knowledge on how the nutritional composition and the antioxidant activity of grapes (both in grape juice and grape pomace) from two traditional winemaking grape varieties, ‘Grenache’ and ‘Cabernet Sauvignon’, are modulated by varying the harvest timing and the sunlight exposure. The large amounts of wine pomace obtained from the winemaking process and the potential market for its use has led us to investigate it in order to explore alternatives that allow for the maximisation of the potential of this byproduct.

## 2. Materials and Methods

### 2.1. Chemicals

Fluorescein, 6-hydroxy-2,5,7,8-tetramethyl-2-carboxylic acid (Trolox), 2,20-diazobis-(2-aminodinopropane)-dihydrochloride (AAPH), 2,2-diphenyl-1-picrylhydrazyl (DPPH), 2,20-azinobis 3-ethylbenzothiazoline-6-sulfonic acid (ABTS•+), Folin–Ciocalteu (FC) reagent, gallic acid (GA), iron (III) chloride hexahydrate (FeCl3∙6H2O), iron (II) sulphate heptahydrate (FeSO4∙7H2O), 2,4,6-tripyridyl-triazine (TPTZ), ferulic acid, hidroxybenzoic acid, and p-coumaric acid were obtained from Sigma-Aldrich, Co. (St. Louis, MO, USA).

### 2.2. Raw Material

Grapes (*Vitis vinifera* cv. ‘Cabernet Sauvignon’ and ‘Grenache’) were obtained from the experimental vineyard of the Faculty of Oenology (Universitat Rovira i Virgili, Tarragona, Spain) located in the Mas dels Frares centre (Constantí, Spain) (41°08′44″ N 1°12′02″ E; Altitude: 60 m; 15 km from the Mediterranean Sea) during the campaign of 2022. Both grape varieties were cultivated under uniform agricultural conditions. They were grown using the espalier method, which involves a vertical trellis system for vine support. The climate is characterised by high ambient humidity (60–70%) with hot, dry summers and mild, wet winters. The soil is deep, composed of calcareous and clayey material, with a pH of 7.95 and a loamy texture. The soil classification is Calcixerept typic according to Soil Taxonomy (USDA) and Calcisol haplic according to the WRB (World Reference Base for Soil Resources) [24]. 

At each sampling, the grapes were inspected to ensure their sanitary condition, which was optimal for both varieties throughout the experiment. Grape samples were immediately carried to the laboratory for the determination of ripening parameters: total soluble solids and pH; frozen samples were used for bioactivity.

### 2.3. Experimental Design

**Scheme 1:**
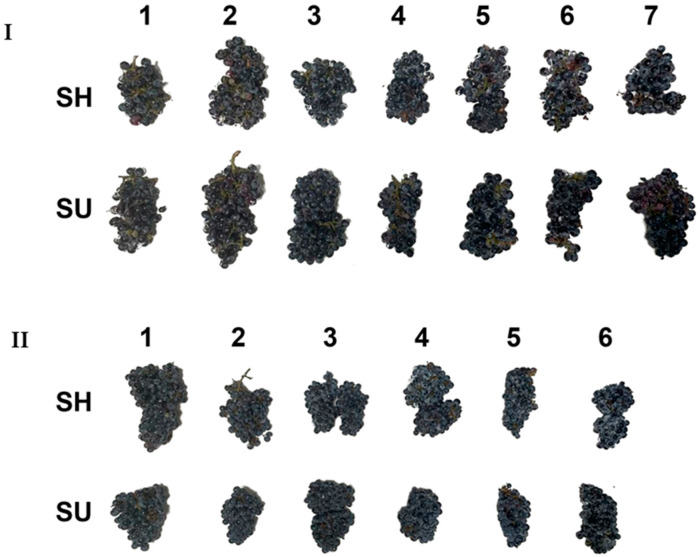
(**I**) ‘Grenache’ (GN) and (**II**) ‘Cabernet Sauvignon’ (CS) grape bunches. (1–7) GN sampling points, from 12th of August to 22nd of September; (1–6) CS sampling points, from 6th of September to 11th of October. Shade (SH, orientation south-west) and sun (SU, orientation north-east) positions.

Two varieties were analysed in this study, ‘Cabernet Sauvignon’ (CS) and ‘Grenache’ (GN). Different harvest times and sunlight exposure were considered as the main parameters of this study. Regarding harvest sampling, several grape bunches (~0.5 kg each sampling) at different times, from 12th of August to 2nd of September for GN (seven sampling points, Scheme 1(I)), sampling from 6th of September to 11th of October for CS (six sampling points, Scheme 1(II)), were harvested. Although both varieties were harvested at the time deemed optimal for winemaking by the oenologist (based on sugar and acidity levels in the berries as well as the organoleptic assessment), a portion of the harvest was intentionally left on the vines for further investigation in this study. It is important to note that the objective of this study does not focus on obtaining wine but rather on the possible attainment of different types of products, with diverse composition and antioxidant properties throughout the grape ripening process.

Comprehensive sampling was performed to ensure the full monitoring of the ripening process (including overripe samples). The exposure of the grape berry to the sun was also evaluated in the study, segregating the samples according to sun (SU, orientation north-east) and shade (SH, orientation south-west) position, in order to evaluate the possible changes in the phenolic composition due to different sun exposures.

### 2.4. Grape Processing Procedure

**Scheme 2:**
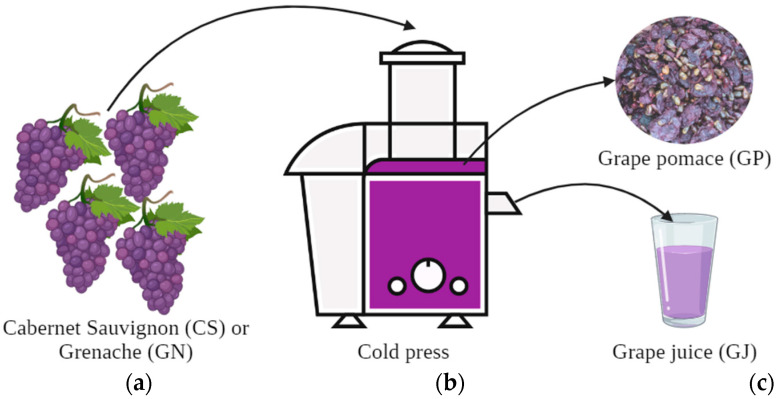
(**a**) Grape fruit (GF); (**b**) processing and (**c**) obtention of grape juice (GJ) and grape pomace (GP).

After each harvest point, the samples were frozen at −20 °C until processing. Before processing, grapes were defrosted at 4 °C overnight and washed using tap water at room temperature (RT) to remove dirt from the surface of the berries. Stems and leaves were removed, and berries were crushed using an automatic BioChef Axis Cold. At the first stage, two fractions were obtained: grape pomace (GP) and grape juice (GJ). The grape pomace and juice were freeze-dried and grounded using a refrigerated mill (Model: IKA M20; IKA-Werke GMBH & Co. KG, Staufen, Germany) until a fine flour was obtained. Flours were sieved to a particle size below 500 µm. All samples were stored at −80 °C until further use (Scheme 2).

### 2.5. Determination of Indicators of Ripening: Total Soluble Solids and pH

After each harvest point, the samples were immediately carried to the laboratory, where a representative number of grapes (different bunches and different positions within the bunch) were crushed and analysed. Analyses of sugar content, measured as total soluble solids (TSS) were conducted at room temperature by using an automatic-temperature-compensation digital handheld refractometer (HI 96801, Hanna Instruments, Smithfield, RI, USA). Prior to each use, the refractometer was calibrated using deionised water. The crystal was thoroughly cleaned with deionised water and wiped dry with cellulose tissues before each new reading, ensuring the accuracy of the measurement.

The pH was measured directly with a portable pH metre with a Micro P portable electrode (7+ series portable pH-metre, XS Instruments, Carpi, Italy). The pH metre was calibrated before analysis.

### 2.6. Proximal Composition

The composition of the grapes (G) and grape pomaces (GP) was determined for all the points tested in the study. First, the Dumas method, 990.03 [25], was used to determine the total protein content using an elemental analyser (LECO Corp., St. Joseph, MI, USA). A petroleum ether extraction (40–60 °C) for 4 h in a Soxhlet extraction unit (AOAC 2005, method 2003.05) [26] was used to determine the total fat content. The moisture content was measured by drying three grams of powdered sample (WB, OH) at 105 °C for 3 h. For ash content, the samples were incinerated at 550 °C for 5 h in a muffle furnace (AOAC 2005, method 923.03) [26]. Carbohydrates were estimated by difference. The total dietary fibre (TDF) content was evaluated using a kit provided by Sigma (TDF100A-1KT, St. Louis, MO, USA), in accordance with the manufacturer’s instructions, based on the AOAC method 985.29. The results were expressed in g 100 g^−1^ of dry matter (d.m.). All analyses were performed in duplicate.

### 2.7. Extracts Preparation

One-gram samples of freeze-dried pomace or juice (GP and GJ) were ground (mesh size 0.5 mm) and extracted with 10 mL of methanol/water (1:1, *v*:*v*; acidified to pH = 2 with 0.1 M HCl) in an orbital shaker (250 rpm, 25 °C) for 30 min. After centrifugation (Model 5810R, Eppendorf, Hamburg, Germany) (2057× *g*, 10 min), the supernatant was collected and filtered (Filter lab paper n. 1249). The methanol/water extraction was repeated three times. After this, the pellet was further extracted with 10 mL of acetone/water (70:30, *v*:*v*) in an orbital shaker (250 rpm, 25 °C) for 20 min and centrifuged (2057× *g*, 10 min). The acetone/water extraction was repeated two times. The five supernatants were pooled, filtered (Whatman 1), and concentrated (Multivapor™ P-12; Buchi, Flawil, Switzerland) until a final volume of 20 mL was reached.

### 2.8. Total Phenol (TP) Content

Folin–Ciocalteu phenol reagent, according to the method described by Slinkard and Singleton [27], was used to determine the total phenol content (TP). A gallic acid standard curve (98–700 μM) was prepared. Standards and sample absorbance were measured at 765 nm using a microplate reader (Fluostar Omega, BMG, Ortenberg, Germany). The results were expressed as μmol gallic acid equivalents (GAE) 100 g^−1^ d.m. All analyses were carried out in duplicate.

### 2.9. Total Antioxidant Activity (TAC)

TAC was measured on extracts using 2,2-diphenyl-1-picrylhydrazyl radical (DPPH), 2,20-azinobis-(3-ethylbenzothiazoline-6-sulfonate (ABTS•+), oxygen radical absorbance capacity (ORAC), and ferric reducing ability potential (FRAP) assays. DPPH and ABTS•+ modified methods were applied on solid samples without previous extraction as quencher methodologies (Q-DPPH and Q-ABTS•+), to evaluate the total antioxidant activity of whole integrated samples. Samples were evaluated in duplicate.

#### 2.9.1. DPPH Radical Scavenging Activity and Q-DPPH Radical Scavenging Activity

The extract-based DPPH assay was carried out as described by Brand-Williams et al. [28], with modifications. A 120 µM DPPH working solution in pure methanol was prepared. In a 96-well microplate, a volume of 25 µL of extracts was mixed with 100 µL of milliQ water and 125 µL of DPPH working solution. The decay absorbance at 525 nm was recorded over 30 min with a microplate reader (Fluostar Omega, BMG, Ortenberg, Germany). Different solutions of Trolox (7.5–240 µM) were evaluated to calibrate a calibration curve. Results were expressed as mg Trolox equivalents (TE) 100 g^−1^ sample. The solid sample-based Q-DPPH method was assayed following the procedure by Serpen et al. [29], with modifications. Ten milligrams of solid samples (<300 µm) were mixed with 30 mL of DPPH working solution (60 µM) prepared in methanol. After incubation at 700 rpm for 30 min (Thermomixer Compact, Eppendorf, AG, Hamburg, Germany), samples were centrifuged at 14,000× *g* for 2 min and the absorbance was measured at 515 nm. The results were expressed as mg of Trolox equivalents (TE) 100 g^−1^ sample.

#### 2.9.2. Oxygen Radical Absorbance Capacity (ORAC)

ORAC assay was performed following a method previously described by Ou et al. [30] with modifications. Phosphate buffer (75 mM, pH 7.4) was used to dilute the Trolox standard curve (7.5–210 μM) and samples. In a black 96-well microplate, 25 μL of sample, Trolox standard, and phosphate buffer as blank were mixed with 125 μL of fluorescein and incubated at 37 °C for 3 min. Subsequently, 25 μL of AAPH solution was added to initiate the oxidation reaction, and fluorescence was monitored for 120 min with a microplate reader (CLARIOstar Plus, BMG, Ortenberg, Germany) using 485 nm excitation and 520 nm emission filters. To obtain the results, the area under the fluorescein decay curve was calculated as a function of Trolox concentration. The data were shown as μmol of TE 100 g^−1^ sample (d.m.).

#### 2.9.3. ABTS•+ Radical Cation Scavenging Activity and Q-ABTS•+ Radical Cation

The scavenging activity of ABTS•+ was measured following the method first described by Miller and Rice-Evans [31], as modified by Martin-Diana et al. [32]. The absorbance was measured at 730 nm. Results were expressed as mg Trolox equivalents (TE) 100 g^−1^ sample. The Q-ABTS•+ method described by Serpen et al. [29], as modified by Martin-Diana et al. [32], was applied to evaluate the direct antioxidant capacity of samples. Ten milligrams of sample was mixed with 30 mL of ABTS•+ working solution. A volume of 3 mL methanol/water (50:50 *v*:*v*) was added to the sample assays to equal the final volume present in the calibration curve run. A calibration curve with Trolox as standard (7.5–240 µM) was used. After 30 min of incubation in darkness, the decay in absorbance was measured at 730 nm. The results were expressed as mg of Trolox equivalents (TE) 100 g^−1^ sample.

#### 2.9.4. Ferric Reducing Antioxidant Power (FRAP)

FRAP was based on the method described by Benzie and Strain [33] with some modifications. To prepare the FRAP working solution, acetate buffer (300 mM, pH 3.6), TPTZ solution (10 mM in 40 mM HCl), and FeCl_3_∙6H_2_O solution (20 mM) were mixed in a 10:1:1 volume ratio. A FeSO_4_∙7H_2_O curve (400–3000 μM) was prepared as a standard. In Eppendorf tubes, 20 μL of the sample and standard or distilled water as blank were mixed with 1.9 mL of FRAP working solution. The tubes were stirred and incubated for 5 min, and the absorbances were measured at 593 nm using a microplate reader (Spectrostar Omega, BMG Ortenberg, Germany). The results were expressed as mmol of Fe Equivalents (FeE) 100 g^−1^ sample (d.m.).

### 2.10. Statistical Analysis

The results were expressed as mean and standard deviation. Analysis of variance (ANOVA) and Duncan’s post hoc tests were carried out to detect differences between mean values. Statgraphics Centurion XVI^®^ software (StatPoint Technologies, Inc., Warrenton, VA, USA) was used to perform the statistical analyses. Analysis of Variance (ANOVA)–Simultaneous Component Analysis (ASCA) was used to decompose the sources of variability influencing the data obtained. ASCA is a multivariate extension of ANOVA, which decomposes the variation in the data into the main effects and their binary combinations according to a predefined experimental design. In this study, two variability factors were considered: (1) on which side of the vine the grapes grow (sun/shade factor), (2) the moment of the maturity process and the interactions between them. ASCA was applied to each individual grape variety for the TP and TAC values in juice and pomace.

## 3. Results and Discussion

### 3.1. Determination of Indicators of Ripening: Total Soluble Solids and pH

An exhaustive sampling procedure was conducted within the collected bunches, involving the selection of one hundred grapes to evaluate grape maturity immediately upon arrival at the laboratory. Table 1I,II show maturity parameters (in terms of total soluble solids (TSS) and pH) for Cabernet Sauvignon and Grenache grapes, respectively.

The trajectory of sugar concentration (TSS) in Cabernet Sauvignon displays an upward trend until the fifth sampling point, with a subsequent decrease attributed to over-ripening and the rainfall of October. It should be noted that the harvest point coincided with point 4, which demonstrates that the viticulturist chose the date wisely, as from that moment, the pH begins to decrease while sugars continue to increase slightly, resulting in the subsequent imbalance that this causes in wine.

On the other hand, the results showed significant differences in both TSS and pH values between samples exposed differently to sunlight (SU/SH), highlighting the effects of sun radiation on grape metabolism and compound accumulation [34]. Regarding pH, differences between SU and SH grapes persisted over time, exhibiting similar values on the optimal harvest day but revealing a higher pH on the SH side for overripe grapes, along with a more substantial increase compared to SU grapes.

For Grenache grapes, which were harvested for vinification between point 4 and 5, similar trends were observed, although the influence of early September rain posed a setback in TSS values. A simultaneous rise in pH due to the rain was also noted, with a parallel evolution on both sides of the vine.

Thus, as shown by the values in Table 1, the evolution of grapes from both varieties over the studied period was as expected, which is a crucial factor for grape composition [35]. Furthermore, considering that different sunlight exposure significantly affects the main grape parameters, the 26 samples collected (comprising two varieties, six or seven harvest days, and two light orientations) exhibit variations that were also reflected in subsequent analyses, mainly in those related to their antioxidant capabilities.

### 3.2. Determination of Proximal Composition

Grenache and Cabernet Sauvignon grape juices (GJ) and grape pomaces (GP) were analysed for proximal compositions at different ripening times and sunlight conditions (Table 2).

The ash content of Cabernet Sauvignon ranged from 2.35 to 2.69 (g 100 g^−1^), and from 3.2 to 4.38 (g 100 g^−1^) for grape juice (GJ) and pomace (GP), respectively. In the case of the Grenache grape variety, the ash content was from 1.63 to 2.90 (g 100 g^−1^) in GJ and from 3.19 to 3.88 (g 100 g^−1^) in GP (Table 2). It is important to mention that the ash content is associated mostly with minerals and, in the case of grapes and grape byproducts (pomaces), the main minerals identified are calcium (Ca), iron (Fe), magnesium (Mg), phosphorus (P), potassium (K), sodium (Na), copper (Cu), and manganese (Mn). Quintaes et al. reported that grape juice and pomace can provide nearly 100% of the recommended daily intake of Cu and Mn and 50% of Ca, Fe, and Mg [36]. Regarding the values found in this study and in accordance with the bibliography [37], these were within the expected range of the ash content, even tending towards low values. Therefore, it can be ensured that the plants did not suffer any metal accumulation that could have affected their metabolism, so the processed GJ and GP contain normal amounts of minerals.

Total dietary fibre (TDF) values of the CS grape observed were between 11.43 and 19.53 (g 100 g^−1^) for juice and between 42.20 and 65.80 (g 100 g^−1^) for pomace. In the case of GN, the TDF content was from 11.43 to 17.22 (g 100 g^−1^) in juice and from 25.90 to 54.0 (g 100 g^−1^) in pomace (Table 2). The values were similar to those reported by San Martín-Hernández et al. [38] who found significantly (*p*-value < 0.05) higher values in Cabernet Sauvignon, as compared to Grenache. In the case of pomace (GP), our values doubled the content found in juice and were in accordance with values reported by Spinei et al. [39] who found 46.17 ± 0.80 g 100 g^−1^ in grape pomace from red grapes; these authors also showed that the TDF content in pomaces were higher in Cabernet Sauvignon than Grenache. Fibres in grapes and grape pomace are composed by insoluble (cellulose, hemicellulose, lignin, etc.) and soluble (pectin, inulin, gums, and mucilage) fractions. Grape pomace is rich in cellulose, hemicellulose, lignin, and pectin and the seeds are richer in fibre than grape skin [40]. But grape skin is high in lignocellulosic complex containing a considerable amount of hemicellulose sugars and pectic substances, as described by Devesa-Rey et al. [41]. Due to its high content in TDF, GP can be an interesting ingredient for novel product formulation to satisfy the increasing demand for high-fibre diets, which have been shown to promote digestive and systemic health [42]. Moreover, the high content of fibre provides GP with excellent properties as a natural hydrocolloid.

The fat content in CS samples resulted in a range from 0.62 to 2.45 (g 100 g^−1^) for GJ and from 5.33 to 7.40 (g 100 g^−1^) for GP. In GN, the fat content ranged from 0.79 to 1.79 (g 100 g^−1^) in GJ and from 2.35 to 6.47 (g 100 g^−1^) in GP. These fat content values were lower in grape juices (below 3%) than in pomaces (below 8%), and at the same range for those reported by Cabernet by other authors (~7.66%) but lower in the case of Grenache [38]. The differences in the fat content of the Grenache may be associated with the size of the seed part, which is responsible for the fat content. The plant material was less than 5 years old, and maybe this explains the smaller size of the seeds and the lower fat content compared to other studies published. The fatty acid profile in grapes has been reported to present antioxidant and anti-inflammatory activities, as well as other therapeutic properties [43]. Mono- and polyunsaturated fatty acids (90%), especially linoleic acid (58–78%), followed by oleic acid (3–15%), are responsible for these bioactive properties [44,45].

The protein content of Cabernet Sauvignon ranged from 0.52 to 3.88 (g 100 g^−1^) in GJ and from 1.0 to 1.32 (g 100 g^−1^) in GP. In the case of Grenache, this macronutrient ranged from 2.11 to 4.77 in GJ and from 5.11 to 6.75 (g 100 g^−1^) in GP. The protein values of GP were lower than those found in the literature. Particularly, a range from 9.8 to 13.8 g of protein per 100 g of sample was reported for pomaces obtained from varieties Primitivo and Cencibel, respectively [46,47].

Total carbohydrate content, including total dietary fibre, was also quantified in Cabernet Sauvignon and Grenache grapes and pomaces. The values in GN were higher than those in CS for juice and pomaces, in contrast with the other parameters evaluated (protein, TDF, fat, and minerals) that showed an inverse trend. CS carbohydrates in GJ were from 85.38 to 92.08 (g 100 g^−1^) and in GP from 73.10 to 76.92 (g 100 g^−1^). Meanwhile, in GN, carbohydrates were from 91.28 to 94.42 (g 100 g^−1^) in GJ and from 74.75 to 78.46 (g 100 g^−1^) in GP.

It can be concluded that no significant differences (*p* > 0.05) due to sunlight exposure or ripening were observed, after carrying out the multifactor ANOVA analysis (Table 3); although, the sunlight factor resulted in lower *p* values than ripening, suggesting that it is a factor with potentially more influence on the proximal composition. On the other hand, the differences due to variety and product type (juice or pomace) were significant for all compositional parameters evaluated: ash, TDF, fat, protein, and carbohydrates.

The differences in the composition of grapes and grape pomaces may vary depending on extrinsic factors such as edaphoclimatic conditions and agronomic practices such as the lack of fertilisation applied during the season [48,49], which could be a possible explanation for the low protein levels found in this study.

### 3.3. Total Phenolic (TP) Content

**Figure 1:**
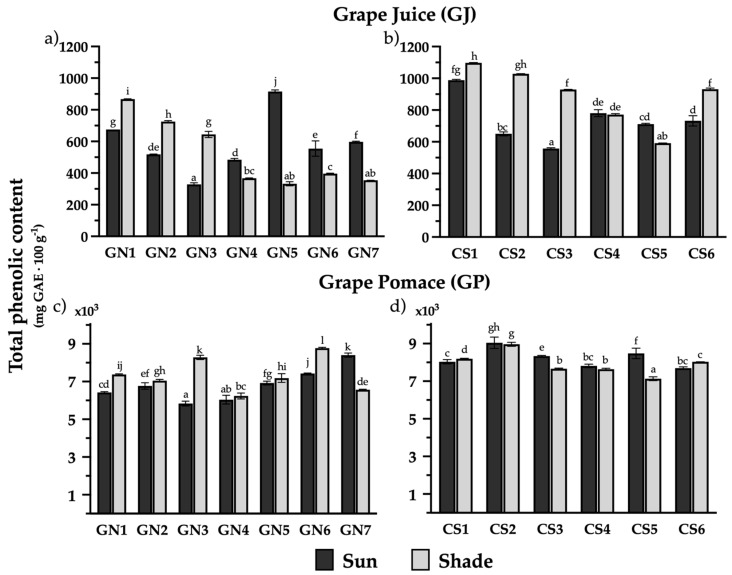
Total phenolic (TP) content for grape juice (GJ) for Grenache (**a**) and Cabernet Sauvignon (**b**) and grape pomace (GP) for Grenache (**c**) and Cabernet Sauvignon (**d**) at different harvest times, for sun and shade exposure. Results were expressed in mg GAE 100 g^−1^ of d.m. Different letters indicate significant differences (*p*-value < 0.05).

The total phenolic (TP) content was measured for Grenache (GN) and Cabernet Sauvignon (CS) over the ripening period, at two different sunlight (sun/shade) conditions (Figure 1).

In grape juice (GJ) samples, the TP values measured ranged between 329 and 916 mg GAE 100 g^−1^ and between 558 and 1097 mg GAE 100 g^−1^, for GN and CS, respectively (Figure 1a,b). The TP content significantly (*p*-value < 0.05) decreased in GJ over the ripening period. A significant (*p* ≤ 0.05) interaction effect of sunlight position (SP) and ripening time (RT) variables was observed; a higher TP content at shade positions during the first three sampling points of the RT was observed for GN and CS; after this, grape juice from Grenache berries growing under sunlight resulted in higher TP values than those of shade positions, with a similar effect observed, except at the last ripening sampling point, in the case of CS samples (Figure 1a,b). The higher values observed at the beginning of ripening can be associated to the reduction in the photosynthetic pathway producing an enhancing of soluble phenols in juice.

The observed values of TP in GJ were in the range described by Garrido and Borges for fresh grape juice (400 to 3000 mg/L) [50], considering that the water content of the juice was in the range of 82–85%.

The TP content was also analysed in the grape pomace (GP) due to the important volume obtained of this byproduct after grape processing. The results showed that the content of total phenols was 100 times higher in pomaces than juices (Figure 1c,d). Grenache showed TP values from 5835 to 8772 mg GAE 100 g^−1^; meanwhile, in Cabernet Sauvignon, the values ranged from 7637 to 9040 mg GAE 100 g^−1^. GN pomace showed a significant (*p*-value < 0.05) increase in TP content during the ripening until the optimum point of harvesting (GN5), regardless of the position of the plant (sun/shade). After the winemaking sampling point (point 5), the grapes with sunlight exposure showed an increase in TP; meanwhile, the grapes harvested from shade had a decrease in the total values. In the case of Cabernet Sauvignon, the behaviour in the pomace did not show a significant trend during the ripening; final values were similar to those of sample point 1. It is important to highlight that most of the grape polyphenols remained in the pomace after the juice process, showing a great opportunity for returning them, within a circular economy strategy, back into the food processing industry [51,52,53].

The values of TP observed in pomace were higher than those reported by other authors (985 to 2122 mg GAE 100 g^−1^), although these differences are probably due to the fact that they studied other grape varieties [54,55]. The results obtained regarding the content of total polyphenols (TP) in the pomace showed no significant differences between the two varieties studied. However, the TP content was dependent on the light position (sun/shade), in the case of Grenache. As it has been reported, more than 45% of TP and total tannins remained in the grape skin pomace, which is the reason for the high content observed in pomace as compared to juice [56].

### 3.4. Total Antioxidant Capacity (TAC)

The antioxidant potential of the different samples was determined. The antioxidant capacity of each extract cannot be assessed by a single method. Indeed, antioxidant measurements are related either to the capacity to transfer a proton (ORAC) or an electron (DPPH or ABTS•+) in order to neutralise radicals [57]. Also, ferric reducing antioxidant power was evaluated to gain further insight into the electron-transfer TAC activity. For this reason, it is necessary to use more than one antioxidant measurement to assess the total antioxidant capacity [58].

**Figure 2:**
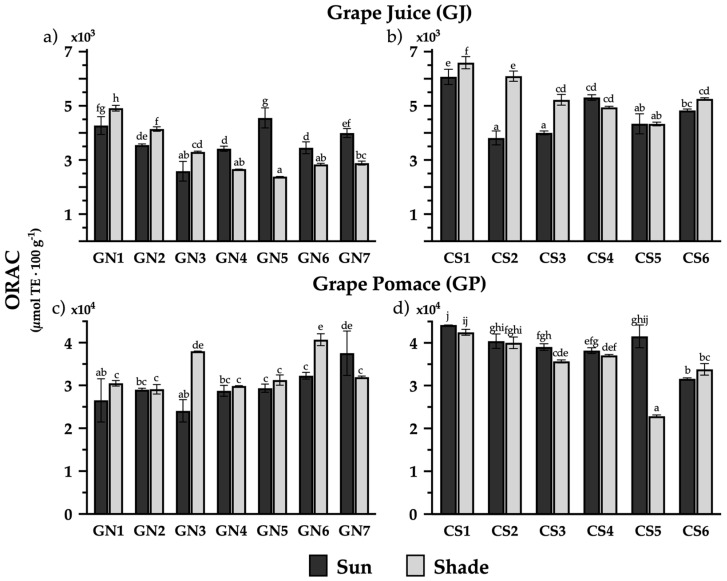
Oxygen radical absorbance capacity (ORAC) values for grape juice (GJ) for Grenache (**a**) and Cabernet Sauvignon (**b**) and grape pomace (GP) for Grenache (**c**) and Cabernet Sauvignon (**d**) at different harvest times, for sun and shade exposure. Results were expressed in mg GAE 100 g^−1^ of d.m. Different letters indicate significant differences (*p*-value < 0.05).

ORAC values observed for GJ were in the range between 2385 and 4915 and between 3814 and 6593 µmol TE 100 g^−1^, for Grenache and Cabernet Sauvignon, respectively, being significantly higher (*p*-value < 0.05) in CS than in GN (Figure 2a,b).

Regarding the effect of ripening, a significant decrease (*p*-value < 0.05) over time was observed in both varieties, results which correlate with those observed in the TP content. As it occurred with TP values, an interaction effect of ripening time and sunlight position was observed, with higher TAC values for samples placed in the shade position at the first sampling points of ripening (1–3). After sampling point 3, GN berries placed in the sun position showed higher ORAC values, and in the case of CS samples, no differences were due to sunlight position after sampling point 4 (Figure 2c,d).

The grape pomace ORAC antioxidant capacity was also evaluated (Figure 2). Values were in the range between 22,955 and 37,540 and between 22,872 and 44,178 µmol TE 100 g^−1^, for Grenache and Cabernet Sauvignon, respectively, being significantly higher (*p*-value < 0.05) in CS than in GN.

**Figure 3:**
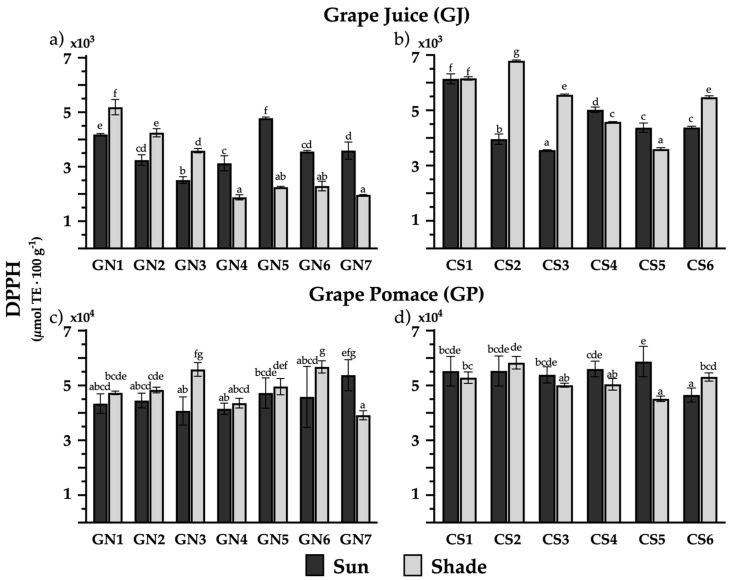
2,2-Diphenyl-1-picrylhydrazyl (DPPH) values for grape juice (GJ) for Grenache (**a**) and Cabernet Sauvignon (**b**) and grape pomace (GP) for Grenache (**c**) and Cabernet Sauvignon (**d**) at different harvest times, for sun and shade exposure. Results were expressed in mg GAE 100 g^−1^ of d.m. Different letters indicate significant differences (*p*-value < 0.05).

The antioxidant activity against the DPPH radical was evaluated in grape juice and pomaces (Figure 3). The values of DPPH in GN and CS juices, for the different ripening times and sunlight position, ranged between 1877 and 5187 and between 3562 and 6795 µmol TE 100 g^−1^, for Grenache and Cabernet Sauvignon, respectively (Figure 3a,b). A reduction in DPPH antiradical activity was observed over ripening time, while the sunlight position had a significant (*p*-value < 0.05) effect, as it did with the TP and ORAC results. In this regard, DPPH values correlated well with those of TP and ORAC, and the interaction effect of ripening time and sunlight position is also observed in the case of DPPH. Good correlations between DPPH and ORAC results, as well as between DPPH and TP, have previously been shown in grapes [56,59].

In the case of grape pomace, the DPPH values ranged between 39,160 and 56,775, and between 45,146 and 58,738 µmol TE 100 g^−1^, for Grenache and Cabernet Sauvignon, respectively (Figure 3c,d). No significant effect on the DPPH results in the grape pomace extracts occurred due to ripening time. In the case of the sunlight position factor, this had the opposite effect in the two varieties. In the case of GN, higher values of DPPH antioxidant capacity were observed for the samples exposed to sunlight position, while in CS, those grapes in the shade position resulted in pomace with higher antioxidant activity.

**Figure 4:**
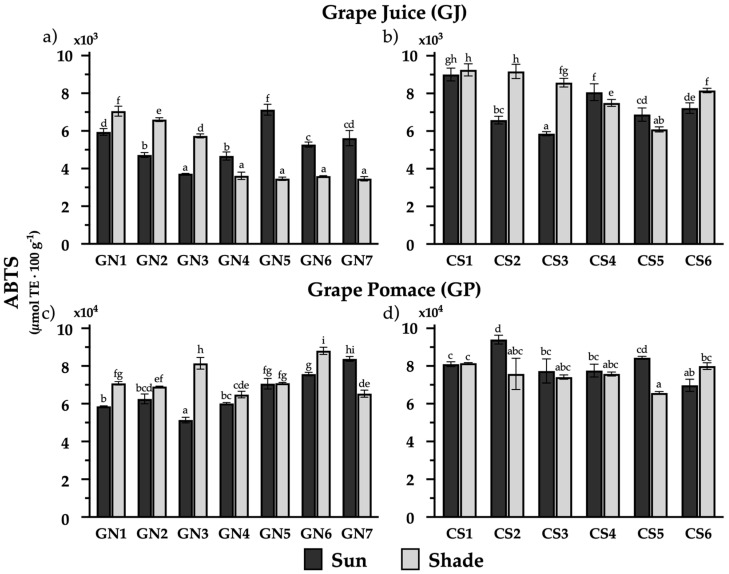
2,2’-azino-bis (3-ethylbenzothiazoline-6-sulfonic acid (ABTS•+) values for grape juice (GJ) for Grenache (**a**) and Cabernet Sauvignon (**b**) and grape pomace (GP) for Grenache (**c**) and Cabernet Sauvignon (**d**) at different harvest times, for sun and shade exposure. Results were expressed in mg GAE 100 g^−1^ of d.m. Different letters indicate significant differences (*p*-value < 0.05).

The ABTS•+ results (Figure 4) followed a similar trend as observed in ORAC and DPPH results. Juice samples had ABTS•+ values between 3460 and 7050 µmol TE 100 g^−1^ and 5857 to 9272 µmol TE 100 g^−1^ for GN and CS varieties, respectively. The highest ABTS•+ antiradical activity of grape juices (GJ) was observed in the first ripening sampling point for grapes under shading conditions, which occurred in both varieties and in the shade position. In the case of grape pomace (GP), no significant effects were observed for ripening time or sunlight direct/indirect incidence (Figure 4). These results are below those found in previous studies, where grape pomace antiradical activity against ABTS•+ resulted in the range from 1 to 5 · 10^5^ µmol TE 100 g^−1^. These differences may be related to differences in extraction methodologies [60].

Regarding the results observed for TAC in extracts with the antioxidant markers (TP, ORAC, DPPH, and ABTS•+), these may be of interest, since the behaviour observed in the antioxidant capacity over the timeframe near optimal ripening could be of use when making decisions in harvest timing. Different harvesting times for grapes at shade positions (they should be harvested when under-ripe) and grapes with higher sunlight incidence (they should be harvested when ripe) may result in maximising the antioxidant activity of the juices obtained, with no detriment of the antioxidant capacity of the valuable byproducts (pomace).

**Figure 5:**
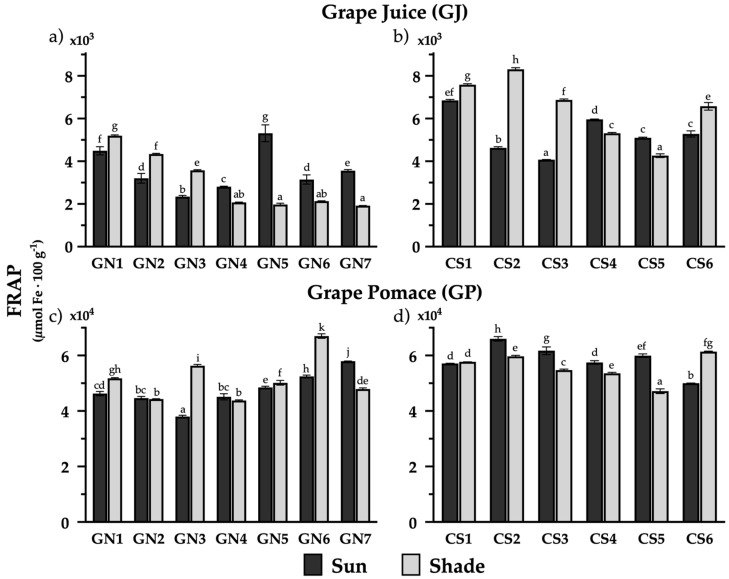
Ferric Reducing Antioxidant Power (FRAP) values for grape juice (GJ) for Grenache (**a**) and Cabernet Sauvignon (**b**) and grape pomace (GP) for Grenache (**c**) and Cabernet Sauvignon (**d**) at different harvest times, for sun and shade exposure. Results were expressed in mg GAE 100 g^−1^ of d.m. Different letters indicate significant differences (*p*-value < 0.05).

The reducing potential against Fe3+ was evaluated for the grape juices of the two varieties (Figure 5a,b). The FRAP results measured were in the range from 381.5 to 729.3 µmol Fe Eq. 100 g^−1^ and from 795.9 to 1449.3 µmol Fe Eq. 100 g^−1^ for GN and CS, respectively. An interactive effect of ripening time and sunlight exposure was observed, as it occurred with the TAC results; in this regard, earlier harvesting times would result in higher FRAP ability of samples under shading conditions, and higher FRAP values would be expected in juices when grapes are harvested at the latest stages of ripening, for both varieties, although this effect is more evident in GN than in CS. The FRAP values observed were higher than those found by other authors for the Cabernet variety, which ranged between 189.51 and 229.66 µmol Fe Eq. L^−1^ [61].

In the case of the grape pomace, FRAP results are shown in Figure 5c,d. These were from 38,015 to 66,993 µmol Fe Eq. 100 g^−1^ and from 47,149 to 65,995 µmol Fe Eq. 100 g^−1^ for GN and CS, respectively. A variety–sunlight position interaction effect was observed, since GN grapes showed higher FRAP values when growing in shading positions, as opposed to CS, where grapes in sunlight positions produced juices with a higher ability to reduce iron. These results are higher than the range reported previously by Rockenbach et al. [60], which found FRAP values for CS pomace from 117.79 to 249.46 µmol Fe Eq. g^−1^. These differences may be due to the redox potentials of the individual phenolic compounds and their structural properties, such as the hydroxylation level and extension of conjugations [62].

**Figure 6:**
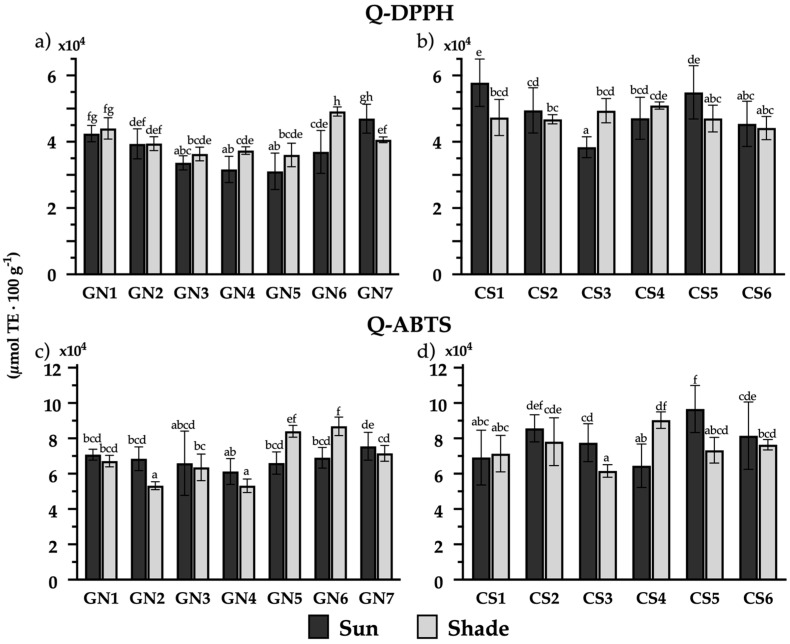
Quencher 2,2-Diphenyl-1-picrylhydrazyl (DPPH) values for grape pomace for Grenache (**a**) and Cabernet Sauvignon (**b**) and quencher 2,2’-azino-bis (3-ethylbenzothiazoline-6-sulfonic acid (ABTS•+) values for grape pomace for Grenache (**c**) and Cabernet Sauvignon (**d**) at different harvest time, for sun and shade exposition. Results were expressed in μmol TE 100 g^−1^ of dry matter. Different letters indicate significant differences (*p*-value < 0.05).

The evaluation of the antioxidant activity through methods applied with extracts presents limitations associated with the extraction conditions. Samples with a high content in bound antioxidant compounds are frequently underestimated in their antioxidant capacity when measured after extraction. For this reason, direct measures (quencher methods) on solid samples (pomace) were carried out in order to evaluate the antiradical ability of the samples against DPPH and ABTS•+ radicals (Figure 6).

Quencher-DPPH results were in the range from 31,090 to 49,140 µmol TE 100 g^−1^ and from 38,375 to 57,818 µmol TE 100 g^−1^ for GN and CS, respectively (Figure 6a,b). In the case of quencher-ABTS•+, the values were from 53,175 to 86,841 µmol TE 100 g^−1^ and from 60,592 to 96,641 µmol TE 100 g^−1^ for GN and CS, respectively (Figure 6c,d). These values were not significantly different from those obtained with extractive methods (DPPH and ABTS•+); the behaviour of the antioxidant capacity over ripening time and as affected by sunlight exposition was similar when evaluated through extracts than with solid samples. In this regard, these results reflect the high extractability of the antioxidant present in the pomace byproduct.

Therefore, through a comprehensive evaluation of all the results obtained, we can assert that there were significant differences among the varieties in relation to the phenolic content and antioxidant properties. The Cabernet Sauvignon variety showed higher total phenolic content and total antioxidant activities than Grenache, which could be related with the higher contents of TDF of CS grape and pomace, as compared with GN. As expected, grape pomace had higher TP content and TAC than juices, since most of the antioxidant compounds are present in the skin and seed, remaining after the processing in the pomace, leading to grape juice with a limited soluble fraction of phenolic content.

Due to the large amount of generated data, Principal Component Analysis (PCA) was applied as a chemometric tool to visualise possible trends in these multivariate datasets. However, the results revealed that due to the variability inherent in the data, no distinct trends could be observed. Nevertheless, given the observed differences in the parameters studied among the different samples, it was considered necessary to determine which factors had a greater impact. Specifically, to study the influence of the considered factors on the values of TP and TAC (DPPH, ABTS•+, ORAC, FRAP, Q-DPPH, and Q-ABTS•+), an ASCA model was built for each grape variety in both the juice and the pomace. The results of the ASCA model are summarised in Table 4.

The ASCA results emphasise the similarities between the two grape varieties, with the maturation process proving to be the most influential factor [35]. Moreover, it can be observed that despite variations in the number of sampling points, both grape varieties encapsulate the variability inherent in an evolving sample, even belonging to the same vineyard. This variability allows for the nuanced modulation of the composition in both grape juice (GJ) and grape pomace (GP) by selecting the collection date. Furthermore, the interactions between factors highlights that both sides of the vine follow a specific maturity process that should also be considered when deciding the collection of the grapes based on their future use.

## 4. Conclusions

The experimental study showed no significant differences associated with either harvesting timing or exposure to sunlight for either of the two studied varieties. However, variety directly affected the protein and total dietary fibre (TDF), since high protein content was observed in juices produced with the GN variety, while GN juices had the highest levels of TDF.

Ripening timing emerged as a critical factor influencing the TP content and TAC of the grape juices, with an interaction effect with sunlight exposure. Grapes that grew in shade positions of the plant may be better harvested earlier (under-ripen), approximately 17 or 35 days before the optimal harvest point, for GN and CS, respectively. This approach would maximise the antioxidant capacity of juices, with no detrimental content in antioxidants in the pomace produced from the juice obtention process. On the other hand, for those grape brunches that grew under direct sunlight incidence, harvesting at the optimal ripening time results in juice with high TAC, while maintaining similar levels of TAC in pomace.

Acknowledging the imperative to minimise waste in the wine industry, all samples were thoroughly characterised in terms of their proximal composition and antioxidant capacity. This comprehensive analysis aimed to investigate the characteristics of the different products obtained, seeking potential applications beyond wine production that allow for the reduction in waste and minimise losses in the viticulture sector.

The results reported may be of interest for a more efficient use of grape products and byproduct valorisation into optimal bioactive juices and pomace-based ingredients and extracts. Understanding the effects of near-optimal harvesting time and sunlight exposure on the antioxidant activity of grape juice and pomace can contribute to the optimisation of decision-making models in grape harvesting. This knowledge contributes to the overarching goal of enhancing sustainability and efficiency in the grape processing industry. These findings show how the control of different factors can contribute to obtaining modified grape-derived products from conventional varieties beyond the wine market.

## Figures and Tables

**Table 1:** Results of Cabernet Sauvignon (I) and Grenache (II) for TSS (total soluble solids, expressed in Brix degrees) and pH. Different letters indicate significant differences between days (*p*-value < 0.05). Asterisks indicate significant differences between SU and SH in the same day (*p*-value < 0.05).

**I**	**Cabernet Sauvignon (CS)**					
	**TSS (ºBrix)**			**pH**		
	**SU**	**SH**	**Significance**	**SU**	**SH**	**Significance**
**CS1**	23.0 ± 0.1	22.0 ± 0.1	a*	3.40 ± 0.01	3.30 ± 0.01	a*
**CS2**	23.9 ± 0.1	23.2 ± 0.2	b*	3.38 ± 0.04	3.26 ± 0.02	a*
**CS3**	23.9 ± 0.1	23.6 ± 0.1	bc*	3.44 ± 0.02	3.47 ± 0.01	b
**CS4**	25.1 ± 0.1	24.8 ± 0.1	d*	3.54 ± 0.01	3.54 ± 0.03	c
**CS5**	26.9 ± 0.1	27.1 ± 0.1	f	3.44 ± 0.02	3.58 ± 0.01	bc*
**CS6**	25.0 ± 0.1	25.9 ± 0.1	e*	3.45 ± 0.03	3.57 ± 0.01	bc*
**II**	**Grenache (GN)**					
	**TSS (ºBrix)**			**pH**		
	**SU**	**SH**	**Significance**	**SU**	**SH**	**Significance**
**GN1**	21.9 ± 0.1	21.9 ± 0.1	a	3.28 ± 0.03	3.25 ± 0.01	a
**GN2**	23.4 ± 0.1	23.1 ± 0.1	b*	3.37 ± 0.01	3.28 ± 0.01	b*
**GN3**	24.4 ± 0.1	24.1 ± 0.1	d*	3.37 ± 0.01	3.40 ± 0.01	c*
**GN4**	23.7 ± 0.1	23.7 ± 0.1	c	3.50 ± 0.04	3.58 ± 0.03	e
**GN5**	23.6 ± 0.1	23.2 ± 0.1	b*	3.52 ± 0.01	3.52 ± 0.01	e
**GN6**	23.8 ± 0.1	23.2 ± 0.1	bc*	3.45 ± 0.04	3.41 ± 0.02	d
**GN7**	24.3 ± 0.1	24.7 ± 0.1	d*	3.43 ± 0.01	3.55 ± 0.02	de*

**Table 2:** Proximal analysis of grape juice (GJ) and grape pomace (GP) for ‘Cabernet Sauvignon (CS)’ (I) and ‘Grenache (GN)’ (II) at different harvest times and optimum times (OP), for sun (SU) and shade (SH) exposure. Values were expressed as g (100 g)^−1^ of dry matter. Different letters in the same row indicate significant differences (*p*-value < 0.05). TDF: total dietary fibre; CH: carbohydrates.

	ASH	TDF	Fat	Moisture	Protein	CH
**GJCS 1 SU**	2.35	15.04	0.73	6.00	2.76	88.16
**GJCS 1 SH**	2.42	20.48	2.26	6.06	3.88	85.38
**GJCS 2 SU**	2.68	15.67	1.02	5.80	2.93	87.57
**GJCS 2 SH**	2.47	24.48	2.45	2.54	3.54	89.00
**GJCS 3 SU**	2.17	18.22	1.44	2.46	3.18	90.75
**GJCS 3 SH**	2.44	19.43	1.23	6.68	3.39	86.26
**GJCS 4 SU**	2.44	14.74	1.55	2.98	2.77	90.26
**GJCS 4 SH**	2.69	15.71	1.27	1.10	2.87	92.08
**GJCS 5 SU**	2.58	18.70	1.29	1.01	3.05	92.07
**GJCS 5 SH**	2.37	17.23	1.09	1.26	3.46	91.82
**GJCS 6 SU**	2.40	18.30	1.65	2.70	2.87	90.38
**GJCS 6 SH**	2.47	16.60	1.44	2.09	2.80	91.20
**GPCS 1 SU**	3.20	53.10	5.33	7.99	1.05	76.92
**GPCS 1 SH**	3.89	61.5	6.80	7.47	1.19	74.40
**GPCS 2 SH**	4.15	59.00	7.10	7.17	1.22	73.95
**GPCS 3 SU**	3.46	53.00	6.21	8.66	1.23	73.99
**GPCS 3 SH**	3.87	56.10	6.90	7.92	1.22	73.69
**GPCS 4 SU**	3.77	56.10	7.40	7.78	1.14	73.92
**GPCS 4 SH**	3.71	53.40	7.20	5.92	1.13	76.11
**GPCS 5 SU**	3.54	49.90	6.63	9.42	1.17	73.10
**GPCS 5 SH**	4.38	48.10	6.09	8.61	1.12	73.92
**GPCS 6 SU**	3.60	42.20	6.58	9.61	1.00	73.96
**GPCS 6 SH**	3.75	54.10	7.30	8.42	1.12	73.53
**GJGN 1 SU**	1.90	17.22	1.37	1.13	0.41	93.02
**GJGN 1 SH**	1.78	13.24	1.69	1.06	0.67	91.28
**GJGN 2 SU**	1.77	11.49	1.13	0.42	0.42	94.09
**GJGN 2 SH**	1.63	15.61	1.79	0.95	0.40	93.12
**GJGN 3 SU**	2.22	12.67	1.21	0.99	0.38	93.21
**GJGN 3 SH**	2.09	19.53	0.86	0.52	0.34	94.42
**GJGN 4 SU**	1.66	14.82	0.83	0.54	0.49	93.93
**GJGN 4 SH**	1.78	13.59	0.79	1.02	0.54	93.07
**GJGN 5 SU**	1.64	16.24	0.85	1.38	0.46	93.28
**GJGN 5 SH**	2.06	19.5	0.88	0.68	0.37	94.05
**GJGN 6 SU**	2.71	11.43	0.94	0.83	0.46	92.67
**GJGN 6 SH**	1.94	11.78	1.02	1.41	0.40	93.13
**GJGN 7 SU**	1.83	12.70	1.01	0.77	0.35	94.21
**GJGN 7 SH**	2.07	15.56	0.97	0.49	0.76	91.69
**GPGN 1 SU**	3.88	54.00	4.12	10.50	1.08	74.75
**GPGN 1 SH**	3.77	49.20	3.38	10.55	0.92	76.55
**GPGN 2 SU**	3.54	40.00	2.86	10.4	0.84	77.92
**GPGN 2 SH**	3.21	35.80	2.38	11.65	0.82	77.65
**GPGN 3 SU**	3.67	45.20	3.99	8.78	1.08	76.81
**GPGN 3 SH**	3.72	36.10	4.29	9.20	1.09	75.98
**GPGN 4 SU**	3.79	25.90	4.58	8.54	1.15	75.9
**GPGN 4 SH**	4.03	51.30	4.63	9.61	1.2	74.23
**GPGN 5 SU**	3.52	49.40	3.71	8.78	1.04	77.49
**GPGN 5 SH**	3.9	52.50	4.27	9.21	1.04	76.12
**GPGN 6 SU**	3.19	33.70	6.47	11.06	0.8	74.27
**GPGN 6 SH**	3.42	38.20	2.9	10.36	0.92	77.59
**GPGN 7 SU**	3.65	44.90	2.35	9.60	0.95	78.46
**GPGN 7 SH**	3.37	38.60	2.88	10.99	0.96	76.75

**Table 3:** Multifactor ANOVA for proximal analysis (ash, total dietary fibre—TDF, fat, protein, and carbohydrates) of factors: variety (Cabernet Sauvignon—CS and Grenache—GN), product (grape juice—GJ and pomace—GP), sunlight (sun—SU and shade—SH) and ripening time (1 to 7 sampling point). *p*-values with * indicate significance between varieties, products, sunlight exposure, and ripening.

Multifactor ANOVA, *p* Values				
	Variety (CS/GN)	Product (GJ/GP)	Sunlight (SU/SH)	Ripening (1–7)
**Ash**	(*p* = 0.003) *	(*p* = 0.00001) *	(*p* = 0.2714)	(*p* = 0.2001)
**TDF**	(*p* = 0.0001) *	(*p* = 0.00001) *	(*p* = 0.1033)	(*p* = 0.9905)
**Fat**	(*p* = 0.00001) *	(*p* = 0.00001) *	(*p* = 0.7373)	(*p* = 0.7423)
**Protein**	(*p* = 0.00001) *	(*p* = 0.0031) *	(*p* = 0.5470)	(*p* = 0.9985)
**Carbohydrates**	(*p* = 0.003) *	(*p* = 0.00001) *	(*p* = 0.4957)	(*p* = 0.6367)

**Table 4:** ASCA results for the TP and TAC values for grape juice and grape pomace, showing the percentage of variance (% Effect) for each factor in each grape variety (CS: Cabernet Sauvignon or GN: Grenache). *: significant effect (*p*-value < 0.05).

Factor	% Effect for CS	% Effect for GN
Sunlight	8.88 *	5.41 *
Grape maturity	42.19 *	42.31 *
Sunlight × Grape maturity	32.26 *	40.95 *
Residuals	16.67	11.33

## Data Availability

The original contributions presented in the study are included in the article, further inquiries can be directed to the corresponding author.
